# Risk factors of knee osteoarthritis: A case-control study

**DOI:** 10.12669/pjms.35.3.277

**Published:** 2019

**Authors:** Nasrin Moghimi, Khaled Rahmani, Ali Delpisheh, Afshin Saidi, Namam Ali Azadi, Abdorrahim Afkhamzadeh

**Affiliations:** 1 *Nasrin Moghimi (MD). Assistant Professor, Department of Rheumatology, Kurdistan University of Medical Sciences, Sanandaj, Iran*; 2 *Khaled Rahmani (PhD). Assistant Professor of Epidemiology, Department of Community Medicine, Kurdistan University of Medical Sciences, Sanandaj, Iran*; 3 *Ali Delpisheh (PhD, Postdoc). Professor of Clinical Epidemiology, Ilam University of Medical Sciences, Ilam, Iran*; 4 *Afshin Saidi (MPH). Liver & Digestive Research Center, Kurdistan University of Medical Sciences, Sanandaj, Iran*; 5 *Namam Ali Azadi (PhD). Biostatistics Department, School of public health, Iran University of Medical Sciences, Tehran, Iran*; 6 *Abdorrahim Afkhamzadeh (MD, MPH). Associate Professor of Community Medicine, Social Determinants of Health Research Center, Research Institute for Health Development, Kurdistan University of Medical Sciences, Sanandaj, Iran*

**Keywords:** Risk factors, Knee osteoarthritis, COPCORD study

## Abstract

**Background and Objective:**

Knee osteoarthritis is one of the most common rheumatologic problems. To investigate risk factors related to the knee osteoarthritis a case-control study was performed using cases diagnosed in the Community Oriented Program for Control of Rheumatic Diseases (COPCORD) study, stage I.

**Methods:**

Using data from the 2012 COPCORD study, stage-I that was conducted in Sanandaj, northwestern of Iran, we runned a case-control study in 2014-2015. Cases were 700 knee osteoarthritis using American College of Rheumatology (ACR) criteria, frequency matched with 700 healthy controls that were randomly selected from the general population.

**Results:**

In multivariate analysis, statistical significant relation was observed between knee OA and some studied factors such as body mass index (P <0.001), lodging (living in highland vs. plain) (P <0.001), type of used toilet (regular vs. toilet) (P <0.001), history of using high-heeled shoes (>3 cm) (P = 0.005), history of knee Injury (P = 0.04), history of lower limb fracture (P = 0.02), Number of pregnancies (P <0.001) and history of pain and swelling (lasting for one months) (P = 0.04).

**Conclusions:**

Living in highland area, using regular toilet, having knee injury and lower limb fracture in the past were most significant associated factors with occurrence of knee osteoarthritis.

## INTRODUCTION

Osteoarthritis (OA) is the most widespread rheumatic illness in human populations leading to chronic pains and severe disabilities worldwide.^1^ Knee is the most common site of the disease and OA, which causes physical disability in elderly.^2^ The OA basically occurs by underlying causes such as previous fractures, bone injuries, infections, rheumatoid arthritis, tumors, congenital and metabolic disorders.^3^,^4^

Community Oriented Program for Control of Rheumatic Diseases (COPCORD) has three stages including determination of prevalence of rheumatic disorders and identification of risk factors; training of primary health-care physicians, paramedical professionals, and improving health care and environmental etiologic researches for rheumatic diseases.^5^ The stage I of Kurdistan COPCORD was conducted during 2012 and its results including prevalence of knee problems has been published in 2015.^6^

Since knee OA is one of the common diseases, is responsible for higher morbidity, particularly in women and in the second half of human life, when the quality of life for patients with knee OA is important issue.^3^,^7^ In terms of importance and necessity of the present study, almost one in five persons aged over 15 years in Kurdistan area, had knee complaints^6^, whilst the corresponding rate was about one in six in Tehran capital as national data.^5^ So, the present study aimed to determine risk factors of knee osteoarthritis in Kurdistan province western Iran.

## METHODS

This case-control study was conducted in Sanandaj city during 2014-2015., the center of Kurdistan province. The ethnicity of study setting is Kurdish, a subgroup of the Caucasians with total population of 311,446 living in 81,380 households.

Cases were those who were diagnosed as knee OA using ACR criteria in Sanandaj COPCORD study.^6^ Controls were also selected from those who were diagnosed healthy and those who had no knee OA. The ACR criteria were used for diagnosing of knee OA, knee pain with at least three out of six criteria in case group. Overall, cases were 700 persons with Knee OA, randomly selected from 1099 knee OA patients diagnosed at the first phase of the COPCORD study. One control (healthy people) was chosen for each case.

The method of data gathering was interview with participants. A valid and reliable questionnaire by the Tehran Rheumatology Research Center was used for data collection.^8^ The interviews were controlled by a rheumatologist advisor and all samples were received a regular quality control visit from the project manager.

Data were analyzed using SPSS version 19. First, univariate analysis was used, then for factors that statistically were significant in univariate analysis and also other variables with P<0.1 were entered in the multivariate analysis, logistic regression model.

## RESULTS

Mean age and standard deviation of case and control groups were 51.8 ± 15.2 and 52.5 ± 15.1 years, respectively (P= 0.4). Sixty percent of participants in each group were female. Mean and standard deviation of investigated quantitative variables in two studied groups and its relation with knee OA were detailed in Table-I.

**Table-I:** Association between potential risk factors (quantitative variables) and knee osteoarthritis.

Variables	Group	Mean	SD	P value
Body Mass	Case	28.8	4.3	<0.001
Index	Control	27.3	4.7	
Daily squatting or crouching (min*)	Case	24.1	2.7	0.7
	Control	23.7	3.3	
Daily chair sitting (minutes)	Case	168.6	24.3	0.6
	Control	170.4	25.2	
Daily kneeling (minutes)	Case	15.5	2.1	0.8
	Control	14.8	1.7	
Using high-heeled shoes (>3cm) in the past	Case	4.6	1.7	0.004
	Control	4.2	1.3	
N. of pregnancies	Case	3.9	4.3	<0.001
	Control	3.0	3.7	
Duration of hypertension (years)	Case	11.3	8.0	<0.001
	Control	6.4	5.8	
Duration of other chronic diseases (years)	Case	17.6	12.6	<0.001
	Control	7.5	7.4	

* Minute.

Association between other qualitative variables and knee OA are presented in Table-II.

**Table-II:** Association between potential risk factors (qualitative variables) and knee osteoarthritis.

Factors	Case, n(%)	Control, n(%)	P value	OR (95%CI)*
Exercise			0.004	1.56 (1.15 – 2.12)
Yes	119 (59.5)	81 (40.5)		
No	581 (48.4)	619 (51.6)		
History of using high-heeled shoes (>3 cm)			0.008	1.42 (1.09 – 1.85)
Yes	163 (57.0)	123 (43.0)		
No	537 (48.2)	577 (51.8)		
Lodging			<0.001	5.24 (4.13 – 6.64)
living in highland area	554 (65.3)	294 (34.7)		
living in plain area	146 (26.4)	406 (73.6)		
Cigarette smoking			0.08	1.3 (0.97 – 1.72)
Yes	124 (55.3)	100 (44.7)		
No	576 (48.9)	600 (51.1)		
History of knee injury			<0.001	0.41 (0.2-0.6)
Yes	60 (69.8)	26 (30.2)		
No	640 (48.7)	674 (51.3)		
History of lower limb fracture			0.001	0.55 (0.30 - 0.80)
Yes	76 (63.3)	44 (36.7)		
No	624 (48.8)	656 (51.2)		
History of pain and swelling (lasting for one month)			0.007	1.34 (1.08 -1.65)
Yes	423 (53.1)	373 (46.9)		
No	277 (45.9)	327 (54.1)		
Menopause			0.032	1.36 (1.0-1.70)
Yes	268 (53.1)	237 (46.9)		
No	161 (45.5)	193 (54.5)		
Familial history of knee problems			<0.001	0.05 (1.1-1.4)
Yes	280 (56.3)	217 (43.7)		
No	360 (44.7	446 (55.3)		
Diabetes			0.004	0.59 (0.41-0.81)
Yes	90 (61.2)	57 (38.8)		
No	601 (48.4)	641 (51.6)		
Hypertension			<0.001	0.60(0.4-0.7)
Yes	177 (59.4)	121 (40.6)		
No	506 (46.9)	574 (53.1)		
Other chronic diseases			0.09	0.80(0.6-1.0)
Yes	198 (53.8)	170 (46.2)		
No	495 (48.3)	529 (51.7)		
Type of toilet use			0.07	1.42 (0.97 – 2.08)
Regular	650 (50.7)	631(49.3)		
Toilet	50 (42.0)	69 (58.0)		

* OR: Odds Ratio, CI: Confidence Interval

Regression logistic results including the values of P value, odds ratio and corresponding 95% confidence intervals for each factor are summarized in Table-III.

**Table-III:** Potential risk factors related to Knee OA (Logistic regression results).

Variable	OR (95%CI)*	P value
Age	1.01 (0.99 – 1.01)	0.1
Body Mass Index	1.09 (1.06 -1.12)	<0.001
Lodging (living in highland vs. plain)	5.67 (4.40 – 7.31)	<0.001
Cigarette smoking	1.22 (0.99 – 1.52)	0.06
Exercise	1.22 (0.86 – 1.73)	0.3
Toilet type use (regular vs. toilet)	2.28 (1.47 – 3.55)	<0.001
History of using high-heeled shoes (>3 cm)	1.53 (1.14 – 2.07)	0.005
History of knee injury	1.74 (1.03 – 2.96)	0.04
Familial history of knee problems	1.26 (0.98-1.62)	0.06
History of lower limb fracture	1.64 (1.07 – 2.53)	0.02
History of pain and swelling (lasting for 1 months)	1.31 (1.01 – 1.70)	0.04
N. of pregnancies	1.08 (1.05 -1.12)	<0.001
Diabetes	1.19 (0.80 – 1.80)	0.4
Hypertension	1.30 (0.95 – 1.80)	0.1
Other chronic disease	1.03 (0.80 – 1.36)	0.8

As shown in Table-III, the chance of knee OA occurrence in persons which lived in highland is significantly 5.67 (4.40 – 7.31) times higher than those lived in plain area. Other factors that had strength relation with knee OA were toilet type use (OR:2.28, CI:1.47 – 3.55), having knee injury in the past (OR:1.74 ,CI:1.03 – 2.96) and persons with lower limb fracture in the past (OR:1.64 ,CI:1.07– 2.53).

## DISCUSSION

In this study potential risk factors related to the knee OA were evaluated among urban population of Sanandaj city, Kurdistan Iran. In fact, the study was the stage III of previous COPCORD that has been designed and conducted in 2012.^6^ According to the study results, although not significant, but the chance of getting knee OA is increased by increasing age. This finding is compatible with the results of previous studies.^8^,^9^

In other previous studies, the female gender was reported as main risk factor for knee OA and other musculoskeletal disease and the difference in gender distribution may be due to sensitivity of cartilage tissue to sex hormones as knee cartilage volume is higher in males than in females^10^-^12^, but we could not asses the relationship between sex and knee OA due to individually matching cases and controls.

Several national^13^ and international studies^14^-^18^ have already indicated the effects of BMI on occurrence of knee OA. In study conducted by El Ayoubi et al. showed that obesity, overweight and area of residence are significant risk factors for knee OA.^19^ The present study confirms this finding (P < 0.001]. Even though in Dahaghin study, this association was borderline.^13^ However, many studies have confirmed the effect of overweight and obesity on knee OA.^7^, ^20^ A recent meta-analysis found the pooled odds ratio for developing knee OA was 2.63 for obese subjects compared to normal weight controls.^21^

This study showed that people, who live in highland areas, suffered more from knee OA. Yoshimura et al have already reported that the risk of knee OA in people who were living in mountainous regions was two-fold higher than those who lived in the forest and beach areas. Many previous studies have suggested climbing, long time downing and upping of stairs, job and life activities as risk factors for osteoarthritis specially knee OA.^22^-^25^ Based on the results, living in highland areas that lead to daily climbing up and down from steep and high places was most important risk factor for knee OA (OR=5.67) in studied subjects. A reason for this association can be that the setting of the study, Sanandaj, was located in the mountainous area and more of the city neighborhoods are not geographically plain, so that people living in highland neighborhoods of the city, inevitably had to daily walk up and down for their activities. Despite of this fact, it seems this important factor, need to be assessed in further studies.

In the present study, smoking was not associated with knee OA (P= 0.06). This finding is very controversial. Although study results of Haq et al is compatible with our finding^26^, but many researchers have reported positive effects of smoking as a protective factor for knee OA.^27^,^28^

The regular toilet using was another significant factor for knee OA. Dahaghin et al. in Tehran found same result and they showed that using the toilet reduces the risk of osteoarthritis,^13^ while our result was not similar to Zeng et al. study.^9^ Indeed, majority of population in our studied area are now using regular toilet due to cultural reasons and this factor can be modified as preventable risk factor with suitable intervention.

History of knee injury and lower limb fracture in the past were two other significant factors for knee OA in the present study, which has already been confirmed.^29^ The lower limb fracture had relationship with knee OA.^7^

### Limitations of the study

Although, the study has many advantages such as adequate sample size, methodological design, controlling for potential confounders using logistic regression model and also presence of expert team, but our results like any other case control design may be affected from recall bias in measuring the exposures and also inability to assess all known and unknown exposures for knee OA such as nutritional and genetic factors.

## CONCLUSIONS

Based on results of this study that was conducted in Sanandaj, the center of Kurdistan province in Iran, living in highland areas, using regular toilet, having history of knee injury and lower limb fracture, higher BMI, using high-heeled shoes in the past, having pain and swelling (lasting for one month) in past years and increasing in the number of pregnancies were recognized as risk factors for knee osteoarthritis.
